# Jellyfish Modulate Bacterial Dynamic and Community Structure

**DOI:** 10.1371/journal.pone.0039274

**Published:** 2012-06-20

**Authors:** Tinkara Tinta, Tjaša Kogovšek, Alenka Malej, Valentina Turk

**Affiliations:** National Institute of Biology, Marine Biology Station, Piran, Slovenia; University of Delaware, United States of America

## Abstract

Jellyfish blooms have increased in coastal areas around the world and the outbreaks have become longer and more frequent over the past few decades. The Mediterranean Sea is among the heavily affected regions and the common bloom - forming taxa are scyphozoans *Aurelia aurita* s.l., *Pelagia noctiluca*, and *Rhizostoma pulmo*. Jellyfish have few natural predators, therefore their carcasses at the termination of a bloom represent an organic-rich substrate that supports rapid bacterial growth, and may have a large impact on the surrounding environment. The focus of this study was to explore whether jellyfish substrate have an impact on bacterial community phylotype selection. We conducted *in situ* jellyfish - enrichment experiment with three different jellyfish species. Bacterial dynamic together with nutrients were monitored to assess decaying jellyfish-bacteria dynamics. Our results show that jellyfish biomass is characterized by protein rich organic matter, which is highly bioavailable to ‘jellyfish - associated’ and ‘free - living’ bacteria, and triggers rapid shifts in bacterial population dynamics and composition. Based on 16S rRNA clone libraries and denaturing gradient gel electrophoresis (DGGE) analysis, we observed a rapid shift in community composition from unculturable *Alphaproteobacteria* to culturable species of *Gammaproteobacteria* and *Flavobacteria*. The results of sequence analyses of bacterial isolates and of total bacterial community determined by culture independent genetic analysis showed the dominance of the *Pseudoalteromonadaceae* and the *Vibrionaceae* families. Elevated levels of dissolved proteins, dissolved organic and inorganic nutrient release, bacterial abundance and carbon production as well as ammonium concentrations characterized the degradation process. The biochemical composition of jellyfish species may influence changes in the amount of accumulated dissolved organic and inorganic nutrients. Our results can contribute insights into possible changes in bacterial population dynamics and nutrient pathways following jellyfish blooms which have important implications for ecology of coastal waters.

## Introduction

Microbes play an important role in biogeochemical cycles in marine ecosystems [1]. There is a set of interactions which occur between specific microbial species and the different components of the marine environment [2]. Bacteria and zooplankton are usually considered as separate groups despite their temporal and spatial co - existence [3]. Zooplankton carcasses are one source of particulate (POM) and dissolved organic matter (DOM) which has been overlooked [4]. The chemical composition of the zooplankton body substantially differs from that of phytoplankton biomass. Due to generally low carbohydrate and lipid storage products, the carbon content of zooplankton is usually lower in relation to nitrogen and phosphorus [5] therefore zooplankton carcasses represent high quality POM [4], [6] and a reservoir of labile organic substrates for marine bacteria [7]–[11]. The zooplankton - derived POM could have major implications for bacterial dynamics and phylotype selection not only on the zooplankton surfaces but also in the surrounding water. Tang et al. [7], [12] have already shown that crustacean zooplankton carcasses enhanced carcass - associated bacterial production and enzyme activity reducing carcass C and N content over time. Furthermore, Tang et al. [12] discovered that bacterial communities on decomposing cladoceran and copepod carcasses rapidly diverged from those in the surrounding water. Communities were similar on different types of zooplankton carcasses, suggesting that carcasses are decomposed by congruent bacterial groups. Therefore, zooplankton carcasses could serve as important microbial microenvironments where rapid and efficient local selection takes place [3].

Gelatinous zooplankton lack a chitinous exoskeleton, consequently their carcasses may decompose faster, compared to non - gelatinous zooplankton [7], [9], [10]. According to results from a few studies the rates of jellyfish – POM decomposition vary from 4–7 days for *Periphylla periphylla*, for *Chrysaora quinquecirrha* 5–8 days [13], and up to 9 days for *Catostylus mosaicus* decomposing on sediments [9]. Proteins represent the most abundant fraction of jellyfish organic matter which is also reflected in their low molar C:N ratio (4.5±1.1, reviewed in 6], which further indicates that jellyfish represent high quality POM for bacteria. As Nagata and Kirchman [14] revealed, the degradation of adsorbed proteins differs among different bacterial strains, which suggests that some bacteria are capable of utilizing adsorbed proteins more efficiently than others. In addition, ectohydrolytic enzyme profiles and activities were found to be highly variable among different bacterial groups [15], [16], therefore changes in bacterial community composition resulting from the introduction of jellyfish as organic matter are to be expected. However, despite the potentially important role of jellyfish biomass as a parameter of bacterial community structure dynamics there have been very few published studies on this topic.

Among gelatinous marine taxa cnidarian jellyfish seem to be most noxious for humans, interfering with several human enterprises [17]. The increase in jellyfish populations seems to be a symptom of the cumulative deterioration of coastal ecosystems, possibly as a consequence of the combined effects of climate and anthropogenic stressors [18]. Despite some controversy [19], several recent studies provide evidence that jellyfish blooms have increased in some coastal areas around the world, and these outbreaks appear to have become more severe and frequent over the past few decades [20]–[23]. Jellyfish blooms can have serious ecological and socio-economic consequences. These include human health threats due to stinging [24], blocking cooling intakes of coastal industry, power plants, and desalinization plants [17], [25], interference with fishing [17], effects on farmed fish [26], acting as vectors of fish pathogens [27], and reductions in commercial fish populations due to predation and competition [28].

Either in their natural or in new environments after introduction, jellyfish are considered to have a significant influence on the ecology of pelagic ecosystems. It has been shown that *Aurelia aurita* s.l. exerts direct predatory pressure on mesozooplankton and microzooplankton populations [29]. There is also evidence of an indirect cascading effect of *Aurelia aurita* s.l. on autotrophic and heterotrophic microbial plankton [30]–[32]. During their life span jellyfish play an important role in providing carbon and nutrients to the microbial loop via several possible pathways: excretion [6], mucus production and release [11], [30], [33], and decaying biomass [8]–[10]. Moreover, Condon and coworkers [11] recently presented evidence that release of DOM into the environment during jellyfish blooms might change the biochemical pathway and biological food web structure in the ambient water. Jellyfish have few natural predators and their abundant carcasses at the termination of a bloom can represent an important source of labile organic substrates and inorganic nutrients for bacteria. After bloom decline, decomposing jellyfish have a strong influence on benthic oxygen and nutrient fluxes [9], [34], and on the microbial community [8], [10]. This idea was supported by the outcome of several previous studies which suggest that variable supply regimes of inorganic nutrients and organic matter together with the composition of the organic matter represent a major force affecting bacterial community composition and perhaps biodiversity [35]–[38].

This study was an extension of our previous research that demonstrated that the decomposition process of dead jellyfish biomass triggered changes in bacterial community and nutrient dynamics [10]. The results from that experiment accompanied with our new results on the elemental and biochemical composition of different jellyfish species [39] raised the question of whether different jellyfish substrates could have major implications for bacterial phylotype selection in the ambient water. One of the predictions of this study was that some bacterial types are possibly more efficient decomposers, which might influence trophic conditions in the surrounding environment. We examined the effects of different jelyfish species (*Aurelia aurita* s.l., *Pelagia noctiluca* and *Rhizostoma pulmo*) on bacterial community dynamics and phylotype selection after the crash of a jellyfish bloom in a simple *in situ* experiment. Changes in microbial abundance and productivity, in bacterial community structure, together with organic and inorganic nutrients were monitored to assess decaying jellyfish-bacteria interactions and nutrient regeneration.

## Results

### Jellyfish Biomass Characteristics, Bacterial and Nutrient Dynamics

Our study was performed in the Gulf of Trieste, the northernmost part of the Adriatic Sea where three scyphozoan species of genera *Aurelia*, *Rhizostoma*, and *Pelagia* recurrently form large blooms. *Aurelia* has been generally present from January to June, while *Rhizostoma* is present all year around with highest abundances in autumn and early winter. Although *Pelagia* is a non resident scyphozoan in the northern Adriatic it formed blooms during the 1970s–1980s and 2000s [40]. The results of basic morphological and biochemical analyses showed high water (95–98%) and low organic matter content, variable wet weights and different elemental composition among species. The average wet weight for *Aurelia* is 137.3±140.7 g, 624.9±42.4 g for *Pelagia* and 2880.8±1406.7 g for *Rhizostoma.* The organic matter content on a dry weight basis includes high salt content from medusa mesoglea and after dialysis of freeze-dried jellyfish tissue all concentrations of nitrogen and carbon were higher (Table 1) [39]. Elemental analyses revealed lower percentages of nitrogen and carbon in *Aurelia* dry weights as compared to *Pelagia* and *Rhizostoma* (Table 1). Carbon to nitrogen ratio (C:N) did not differ much between jellyfish, however it was lower for *Pelagia* (4.0±0.1) compared to *Aurelia* and *Rhizostoma* (4.8±0.1 and 4.6±0.1, respectively) (Table 1). Correspondingly, our preliminary analyses of these scyphomedusae protein contents showed a high percentage of proteins in dialyzed, salt - free samples and slight differences in concentrations between species (Table 1). The lowest protein content was measured in the samples of *Aurelia* (17.7±3.5) and was on average two times lower compared to *Pelagia* (33.1±5.7) and *Rhizostoma* (29.3±4.8).

**Table 1:** Percentages of nitrogen, carbon, C:N ratio and protein content (±SE) of dry weight in non - dialyzed and dialyzed (salt - free) samples of three jellyfish species.

	Non - dialyzed samples			Dialyzed samples			
Jellyfish species	% N	% C	C:N ratio	% N	% C	C:N ratio	% Proteins
*Aurelia aurita* s.l.	0.46±0.1	1.81±0.2	4.6±0.1	7.28±0.6	30.23±2.8	4.8±0.1	17.7±3.5
*Pelagia noctiluca*	2.06±0.2	6.87±0.7	3.9±0.1	11.43±1.0	39.29±2.5	4.0±0.1	33.1±5.7
*Rhizostoma pulmo*	2.56±0.3	9.62±1.2	4.4±0.1	10.51±0.4	41.12±1.4	4.6±0.1	29.3±4.8

In our experiment we assessed the subsequent utilization of jellyfish biomass by the ambient bacterial community in enclosures and compared it to the control seawater without any addition. An equal amount of jellyfish biomass (12.5 g (w/w) per liter, final concentration) was added into enclosures filled with GF/F pre- filtered seawater collected in spring during a bloom of jellyfish. The enclosures were marked as A for *Aurelia*, P for *Pelagia,* R for *Rhizostoma* treatment (A, P and R used subsequently in the text) and C for the enclosure with GF/F pre - filtered seawater without any addition. Changes in bacterial abundance, productivity and community structure were monitored, and dissolved protein as well as inorganic nutrients concentrations were selected to follow the remineralization of jellyfish biomass (for details see Materials and Methods).

**Figure 1:**
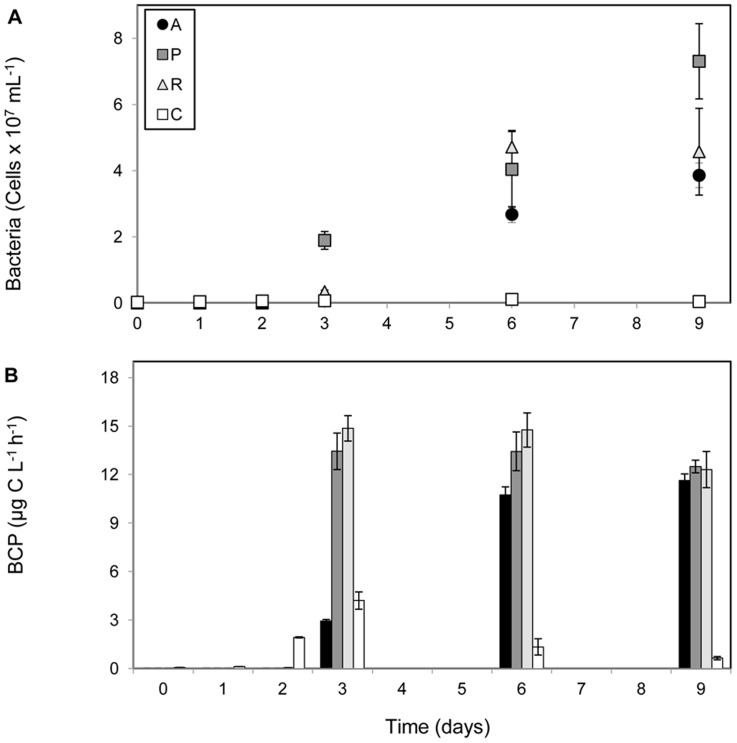
Response of bacterial abundance (A) and production (B) to different jellyfish substrate additions during the enrichment experiment. A - *Aurelia*, P - *Pelagia*, R - *Rhizostoma*, C - control.

The addition of jellyfish biomass had a pronounced effect on bacterial community dynamics as reflected by increases in abundance, growth rates and shifts in community composition in all treatments enriched with jellyfish. After the initial 3 day lag phase bacterial abundance significantly increased from 3.3±0.8×10^5^ to 3.2±0.5×10^7^ cells mL^−1^ by the end of the experiment (Fig. 1A; Table S1). The increase of bacterial abundance was significantly higher in all jellyfish treatments as compared to the control treatment (Fig. 1A; Table S1; ANOVA, Pr(>F) = 0.00657). During the exponential growth phase the bacterial growth rates were 0.46 d^−1^, 1.1 d^−1^and 1.49 d^−1^ in A, R and P treatments, respectively. At the same time the bacterial growth rate was only 0.1 d^−1^ in the control treatment. Bacterial carbon production (BCP), measured using the ^3^H-leucine incorporation method, was also significantly higher in all jellyfish treatments (on average 11.8 µg C L^−1^ h^−1^), compared to the control (Fig. 1B; Table S2; ANOVA, Pr(>F) = 7.5×10^−14^; Tukey HSD test, P = 0).

**Figure 2:**
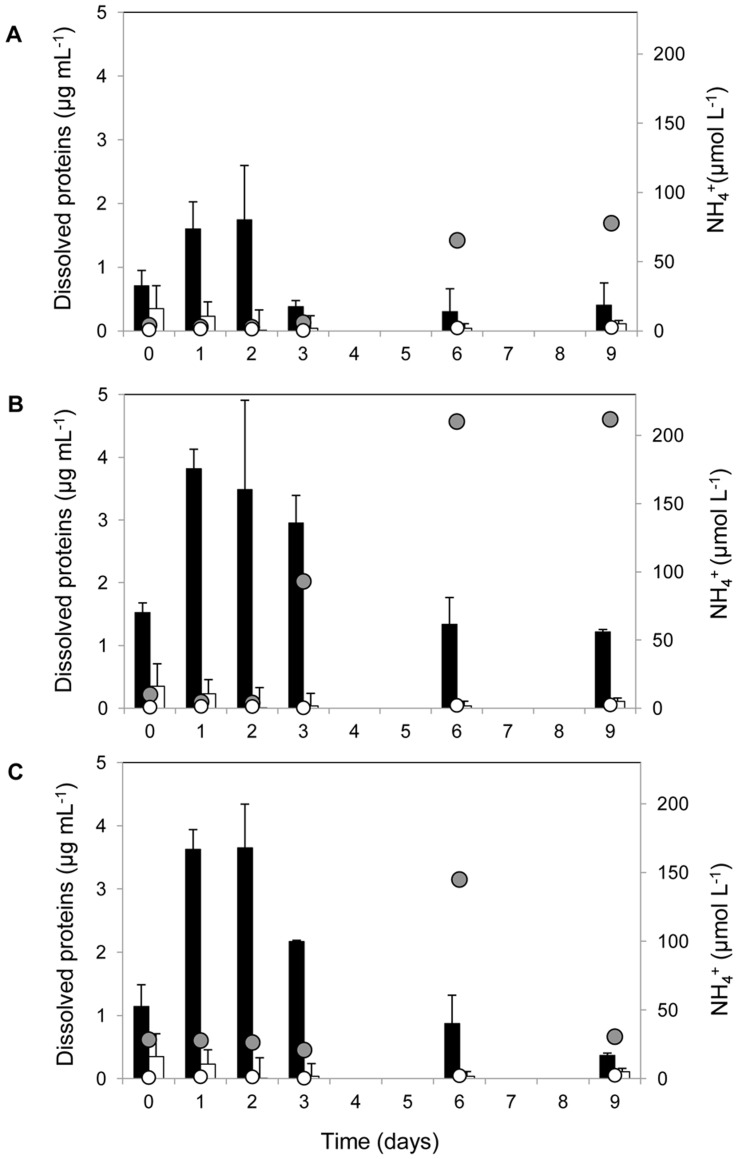
Changes in dissolved protein and ammonium (NH_4_^+^) concentrations during the enrichment experiment. (A) treatment with *Aurelia*, (B) treatment with *Pelagia*, (C) treatment with *Rhizostoma*; ▪ dissolved protein concentration in jellyfish treatment □ dissolved protein concentration in control; • NH_4_^+^ concentration in jellyfish treatment ○ NH_4_^+^ concentration in control.

Over the first 3 days of the experiment, POM decreased by 50–62% (Table 2). At the same time increases in dissolved protein concentrations were recorded in all jellyfish treatments, probably due to the gradual release of proteins from decomposing biomass (Fig. 2A–C). After this initial increase the concentration of dissolved proteins decreased by the end of the experiment at rates of 0.13 µg protein mL^−1^ day^−1^ in A, 0.28 µg protein mL^−1^day^−1^ in P and 0.38 µg protein mL^−1^ day^−1^ in R. The overall dissolved protein decrease was 68% in P, 77% in A and 90% in R (Fig. 2A–C). In the control the POC/PN and dissolved protein concentrations remained significantly lower compared to jellyfish treatments throughout the experiment (Fig. 2A–C; ANOVA, Pr(>F) = 3.9×10^−5^). By the end of the experiment refractory POM ranged from 15% (A treatment) to 50% in P and R treatments (Table 2).

**Table 2:** Particulate organic carbon (POC), particulate nitrogen (PN) and C:N ratio measured at different time intervals during the enrichment experiment.

	POC (mg L^−1^)				PN (mg L^−1^)				C:N ratio			
Day	A	P	R	C	A	P	R	C	A	P	R	C
0	20.3	56.3	49.5	0.4	5.1	15.4	13.3	0.0	4.6	4.3	4.4	/
3	13.5	31.1	28.2	1.2	3.9	3.9	6.0	0.3	4.1	9.2	5.5	4.5
6	9.1	25.9	24.1	1.3	2.0	5.8	5.0	0.8	5.4	5.2	5.6	1.9
9	8.3	21.0	37.4	1.1	2.0	2.3	7.8	0.2	4.7	10.7	5.6	5.2

A - Treatment with *Aurelia.*

P - Treatment with *Pelagia.*

R - Treatment with *Rhizostoma.*

C - Control.

The addition of jellyfish biomass changed dissolved inorganic and organic pool within the enclosures. Ammonium concentration (NH_4_^+^) increased with time in all treatments with jellyfish biomass (Fig. 2A–C; Table S1) and was significantly higher compared to the control (Fig. 2A–C; ANOVA, Pr(>F) = 2×10^−5^; Tukey HSD test, P<0.007). The ammonium accumulation rate expressed as µmol of NH_4_^+^ per gram of added jellyfish per day was 0.82 µmol g^−1^ day^−1^ in A, 1.6 µmol g^−1^ day^−1^ in R and 2.7 µmol g^−1^ day^−1^ in P. Over the first 2 days of the experiment the dissolved organic and inorganic phosphorous concentration increased in all jellyfish enriched treatments, and the orthophosphate concentration remained fairly high until the end of the experiment, compared to the control (data not shown).

### Bacterial Community Structure Dynamics

Changes in bacterial and nutrient dynamics due to the addition of jellyfish biomass were accompanied by shifts in bacterial community structure. During our experiment bacterial community structure was followed using two different approaches: by culture independent genetic analysis using denaturing gradient gel electrophoresis (DGGE) and 16S rRNA clone libraries, and by 16S rRNA sequence analysis of bacterial strains isolated from ZoBell agar.

**Figure 3:**
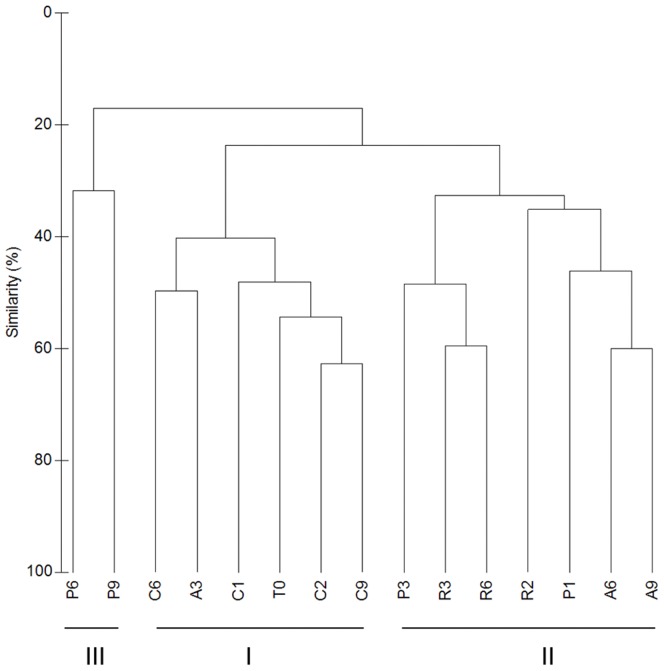
DGGE similarity dendrogram from all sampling time points during the enrichment experiment. Samples collected at the beginning of the experiment (T0), on days 1, 2, 3, 6 and 9 in the control (C1, C2, C6, C9), on days 3, 6 and 9 from the treatment with *Aurelia* (A3, A6, A9), treatment with *Pelagia* (P1, P3, P6, P9) and treatment with *Rhizostoma* (R2, R3, R6). The similarity dendrogram was constructed from DGGE banding patterns using the Bray - Curtis coefficient; clustering was performed using the UPGMA method. I - cluster I, II - cluster II, III - cluster III.

DGGE analysis of 16S rRNA PCR products was used to track changes in bacterial community composition from the beginning (T0) to the end of the experiment (day 9 - T9) in the jellyfish enriched treatments (A, P and R) as well as in the control (C). By comparing all sample fingerprints we observed that bacterial community composition changed over time in the enclosures with jellyfish addition, while the structure of the bacterial community in the control did not diverge much from the starting community. The similarity dendrogram constructed on the basis of the DGGE banding pattern indicates three separate clusters (Fig. 3). The first cluster (Fig. 3–I) consists of the initial seawater community (T0) and the communities isolated from the control, treatment containing seawater without any addition, throughout the experiment (C1, C2, C6 and C9). Samples grouped in one sub - cluster (T0, C1, C2 and C9 communities) had slightly less similarity to a second sub - cluster which contains bacterial communities from the control (C6) and the treatment with *Aurelia* on day 3 (A3). The other two clusters (Fig. 3–II and III) consist of communities from jellyfish enriched treatments (A, P and R). Cluster II divides into two sub-clusters; one consists of the P3, R3 and R6 communities, from which R3 and R6 group together, and the other which consists of R2, P1, A6 and A9 (Fig. 3–II). Cluster III consists strictly of communities from P treatment from the later sampling time points (Fig. 3–III).An interesting observation from cluster analyses is that the bacterial community from the treatment with *Aurelia* at the beginning of the experiment was more similar to the initial ambient seawater population, since the sample A3 grouped in the same cluster as T0 and the control communities, while the communities from treatment P and R on day 3 (P3 and R3) grouped in a separate cluster. This also corresponds to the differences in the time course of the bacterial abundance and productivity dynamics between treatments (Fig. 1). Furthermore, the addition of different jellyfish biomass enhanced the growth of different bacterial community members, since the communities exposed to different jellyfish group into separate clusters (Fig. 3).

**Figure 4:**
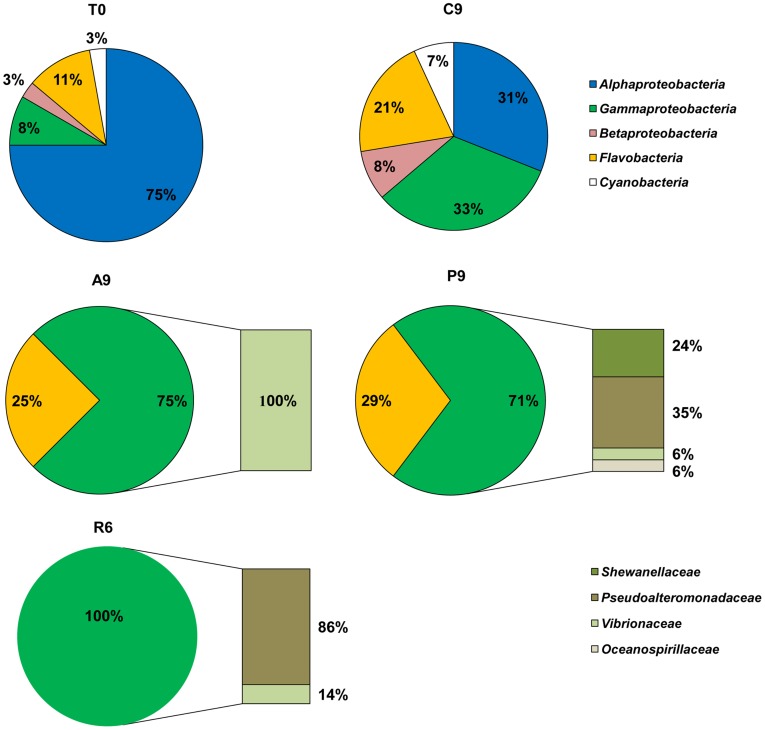
The distribution of phyla in each 16S rRNA library (% of clones) from different treatments. DNA extraction from the sample at the beginning of the experiment (T0), on day 9 from the control (C9), on day 9 from the treatment with *Aurelia* (A9) and treatment with *Pelagia* (P9), and on day 6 from the treatment with *Rhizostoma* (R6).

Analysis of the 16S rRNA gene clone libraries confirmed DGGE and the clustering analysis results and gave us further insights into changes in bacterial diversity (Fig. 4). The initial seawater bacterial community was dominated by unculturable *Alphaproteobacteria* (75%) (almost exclusively affiliated with the SAR11 clade), and a smaller percentage of clones which belonged to *Flavobacteria* (11%), *Gammaproteobacteria* (8%), *Betaproteobacteria* (3%) and *Cyanobacteria* (3%) (Fig. 4 -T0). After 9 days of the experiment the composition in the control treatment remained similar, but with a lower percentage of *Alphaproteobacteria* (31%), and a higher percentage of *Gammaproteobacteria* (33%), *Flavobacteria* (21%), *Betaproteobacteria* (8%) and *Cyanobacteria* (7%) (Fig. 4 - C9). On the other hand, in the jellyfish treatments A, P and R, we observed a reduction in the diversity of the bacterial community by the end of the experiment (A and P - day 9, R - day 6) (Fig. 4: A9, P9, R6). The unculturable *Alphaproteobacteria*, which dominated the community in the T0 library, diminished or was completely absent with time in the jellyfish enriched treatments and were replaced by culturable *Gammaproteobacteria*. In the A9 clone library *Gammaproteobacteria* represented 75% of all bacterial clones, which exclusively belong to the *Vibrionaceae* family, the rest of the clones (25%) were *Flavobacteria* (Fig. 4 - A9). Also in the P9 library *Flavobacteria* represented 29% of clones and again the community was dominated by *Gammaproteobacteria*, which represented 71%, but were different and more diverse compared to A9, with representatives of *Pseudoalteromonadaceae* (35%), *Shewanellaceae* (24%), *Vibrionaceae* (6%) and *Oceanospirillaceae* (6%) (Fig. 4 - P9). The R6 library was composed only of *Gammaproteobacteria*, from which 86% affiliated with *Pseudoalteromonadaceae* and 14% with the *Vibrionaceae* family (Fig. 4 - R6).

When comparing bacterial community composition from the beginning of the experiment (T0) to the communities towards the end of the experiment we observed that, in all treatments, the bacterial population had shifted from unculturable to culturable species known to be associated with different rich organic substrates (Table S3).

During our experiment the bacterial community structure was also followed using 16S rRNA sequence analysis of bacterial colonies isolated from ZoBell agar. 16S rRNA sequences of bacterial isolates from all treatments (A, P, R, C) throughout the experiment, revealed that the culturable bacterial community predominantly affiliated with *Gammaproteobacteria*, dominated by *Vibrionaceae* and by *Pseudoalteromonadaceae* (Table 3). The closest relatives to the 16S rRNA sequences of bacterial colonies from all treatments isolated during the enrichment experiment were similar to bacterial isolates known to be associated with different sea animals, while the closest relatives of bacterial isolates from seawater were similar to known sequences of seawater bacteria, with one exception, *Vibrio splendidus* (Table 3) which was isolated from sea animals.

**Table 3:** List of 16S rRNA sequences of bacterial colonies isolated during the enrichment experiment with high similarity (>99%) to sequences previously reported.

Treatment	Isolate	Acc. No.	Closest relative in GeneBank, Acc. No.	Family	Isolation source
A2	JELLYFISH_Au_A02	JQ432576	Vibrio tasmaniensis strain Mj28, GQ455006	*Vibrionaceae*	Spider crab
A3	JELLYFISH_Au_A03	JQ432577	Vibrio sp. Da4, AF242272	*Vibrionaceae*	Sea urchin
A4	JELLYFISH_Au_A04	JQ432578	Vibrio sp. W-10, DQ923444	*Vibrionaceae*	Sea water
A5	JELLYFISH_Au_A05	JQ432579	Vibrio splendidus isolate PB1-10rrnM, EU091337	*Vibrionaceae*	Atlantic halibut fry
A6	JELLYFISH_Au_A06	JQ432580	Vibrio splendidus strain Mj33, GQ455009	*Vibrionaceae*	Spider crab
P1	JELLYFISH_Pn_P00_1	JQ432571	Psychrobacter nivimaris strain KOPRI24925, EF101544	*Moraxellaceae*	Marine algae
P2	JELLYFISH_Pn_P02	JQ432572	Vibrio sp. B69, FN295820	*Vibrionaceae*	Bryozoa
P3	JELLYFISH_Pn_P03	JQ432573	Vibrio tasmaniensis strain Mj28, GQ455006	*Vibrionaceae*	Spider crab
P4	JELLYFISH_Pn_P04	JQ432574	Vibrio sp. 300108-15, EU862329	*Vibrionaceae*	Fish
P5	JELLYFISH_Pn_P05	JQ432575	Vibrio splendidus isolate PB1-10rrnG, EU091331	*Vibrionaceae*	Atlantic halibut fry
R2	JELLYFISH_Rp_R02	JQ432567	Vibrio splendidus isolate PB1-10rrnA, EU091325	*Vibrionaceae*	Atlantic halibut fry
R3	JELLYFISH_Rp_R03	JQ432568	Psychrobacter sp. 4Dc, HM771256	*Moraxellaceae*	Sea water
R4	JELLYFISH_Rp_R04	JQ432569	Pseudoalteromonas sp. B199b, FN295769	*Pseudoalteromonadaceae*	Bryozoa
R5	JELLYFISH_Rp_R05	JQ432570	Pseudoalteromonas sp. KOPRI 25444, GU062514	*Pseudoalteromonadaceae*	Unknown
C2	JELLYFISH_C_C00_2	JQ432581	Idiomarina loihiensis strain MAH1, FM179981	*Idiomarinaceae*	Sea water
C1	JELLYFISH_C_C01_1	JQ432582	Vibrio splendidus strain Mj82, GQ455013	*Vibrionaceae*	Spider crab
C1	JELLYFISH_C_C02_1	JQ432583	Vibrio splendidus strain Mj33, GQ455009	*Vibrionaceae*	Spider crab
C2	JELLYFISH_C_C02_2	JQ432584	Zunongwangia profunda SM-A87, CP001650	*Flavobacteriaceae*	Sea sediment
C3	JELLYFISH_C_C03_2	JQ432585	Vibrio tasmaniensis strain Mj217, GQ454976	*Vibrionaceae*	Spider crab
C4	JELLYFISH_C_C04_1	JQ432586	Pseudoalteromonas marina strain DHY3, GU198498	*Pseudoalteromonadaceae*	Sea water
C5	JELLYFISH_C_C05	JQ432587	Pseudoalteromonas marina strain S411, FJ457131	*Pseudoalteromonadaceae*	Sea water

## Discussion

Our microscopic observations, results from 16S rRNA clone libraries of the total bacterial community and colony forming bacteria analyses, showed that some bacterial phylotypes are conceivably more efficient jellyfish biomass decomposers, forming microenvironments where rapid and effective breakdown takes place. To our knowledge only one study has been performed thus far showing that the degree of changes in bacterial biodiversity depends on the types of jellyfish biomass involved [8]. According to our results, increase in bacterial abundance resulted from the growth of *Gammaproteobacteria* and *Flavobacteria*, while the growth of *Alphaproteobacteria* (mostly the SAR11 clade), which dominated (75%) the community in the initial seawater population, was inhibited (Fig. 4). In contrast, community composition in the control did not change, although the percentage of *Alphaproteobacteria* decreased. This suggests that the possible ‘bottle effect’ was minimal. *Gammaproteobacteria* and *Flavobacteria* are known to be dominant particle colonizers [41], [42], capable of degrading high - molecular weight organic compounds [43], due to their genes encoding hydrolytic enzymes having a preference for polymeric carbon sources and a distinct capability for surface adhesion [43]–[45]. In contrast the representatives of SAR11 clade, which were dominant in the initial seawater (T0) clone library, may be more important to the flux of low - molecular weight monomers than to that of high - molecular weight polymers. In addition, SAR11 were typically responsible for a greater portion of amino acid and glucose assimilation than of protein assimilation [38]. While the diversity in the control at the end of the experiment was not reduced and remained similar to initial seawater bacterial community structure, we observed a reduction in the diversity of bacterial communities exposed to jellyfish biomass. In all clone libraries retrieved from the jellyfish treatments at the end of the experiment *Gammaproteobacteria* represented from 71 up to 100% of the bacterial community. However, there were differences in the percentages of different family representatives inside *Gammaproteobacteria* between jellyfish enriched treatments. This also explains the observation that they formed separate clusters in the dendrogram constructed on the basis of the DGGE banding pattern. While the *Aurelia* treated community was dominated by *Vibrionaceae*, and the *Rhizostoma* treated community was composed of *Pseudoalteromonadaceae* (86%) and *Vibrionaceae* (14%), the *Pelagia* treated community was the most diverse of them all, with family representatives from the *Alteromonadales* order - *Pseudoalteromonadaceae* (35%), *Shewanellaceae* (24%) and *Oceanospirillaceae* (6%) and from the *Vibrionaceae* family (6%). *Vibrionaceae* are rarely found in clone libraries from environmental samples and represent only a minor fraction of total bacterioplankton [46], [47]. However, they were found in high abundance in eutrophic coastal waters and especially in association with different marine organisms (corals, fish, sponges, shrimp, seagrass and zooplankton) [48]. Furthermore, it was shown that by adding organic substrates to the water, *Vibrionaceae* rapidly respond and become dominant in the bacterial community, suggesting that their high rRNA content enables this accelerated response and allows them to grow rapidly and outcompete other members of the bacterial community [49]. In our study, the absence of *Vibrionaceae* in 16S rRNA clone libraries in the initial seawater and control treatments (T0 and C9) and the fact that jellyfish used in the experiment were not washed before treatment, suggest that these bacteria were introduced into experimental bottles with the jellyfish. Among marine bacteria *Vibrionales* together with *Alteromondales* are known as important producers of antibiotics and inhibitory compounds which might reduce the number of other community members, e.g. *Alphaproteobacteria*. It was suggested that this strategy accounts for the microscale variations in competing bacterial populations and that antagonistic activity was more common among particle - associated bacteria than among free - living ones [50]. This is further supported by our observation that bacterial community composition had shifted from unculturable to culturable species (Table S3). The closest relatives of our bacterial clones and isolates were isolated from living sea animals (as hosts for those bacterial species), detritus, sea sediment or even artificial surfaces submerged in seawater (Table 3, Table S3).

In many coastal and semi - enclosed areas (fjords, bays, and estuaries) gelatinous zooplankton are able to bloom and achieve enormous biomasses (tones per km^−2^) [18], [51]. Our study focuses on the most common bloom - forming species in the Mediterranean such as scyphozoans belonging to the genera *Aurelia*, *Pelagia*, and *Rhizostoma.* Wavelet analysis showed that the periodicity of occurrence of *Aurelia* increased in the northern Adriatic [40], and there are some areas where the population of *Aurelia* persists throughout the year, such as the oligotrophic coastal lake Veliko Jezero (Mljet, southern Adriatic Sea) [52]. In contrast, holoplanktonic *Pelagia* is characteristically found in open water, and large numbers of smaller sized medusa are driven into areas with favourable conditions that stimulate growth and reproduction and promote longer retention. Massive die off will produce abundant particles of jellyfish organic matter and microbial hot spots to fuel bacterial production and nutrient regeneration. In our study and in a few others it was shown experimentally that gelatinous detritus originating from jellyfish can be decomposed within a week by bacterial activity [8], [10]. Jellyfish biomass may decompose within the water column or on the benthos, depending on sinking rates and the depth of the water column as well as environmental conditions [53]. Due to their high POC/PN and protein content, nutrient recycling after decomposition of these blooms cause large accumulations of inorganic nutrients to be released into the environment. In our treatments, the peaks of organic nutrient release were followed by significant bacterial growth and an important flux of inorganic nutrients, with an NH_4_^+^ efflux of 0.67 µmol g^−1^ day^−1^ in the treatment with *Aurelia*, which is comparable to our previous results [10]. In the *Rhizostoma* and *Pelagia* treatments, the rate of NH_4_^+^ flux was even higher, which might be a result of the different elemental and biochemical composition of different jellyfish species [39]. Jellyfish biomass as a source of dissolved inorganic and organic phosphorous could be of considerable importance in phosphorous limited areas such as the Gulf of Trieste (northern Adriatic Sea) [54] and the entire Mediterranean Sea [55].

Our results show that jellyfish biomass is highly bio-available to ‘jellyfish - associated’ and/or ‘free - living’ heterotrophic bacteria that can rapidly decompose it resulting in inorganic nutrient release. Despite the small number of direct experimental data, the impact of the jellyfish - bacteria link could be significant especially in areas where jellyfish attain very high abundances. This may become even more important in the future, as recent analyses indicate an increasing trend from the 1950s in 62% [51] of Large Marine Ecosystems studied.

## Materials and Methods

### Sampling and Jellyfish-enrichment Experiment Set Up

Sampling of jellyfish *Aurelia aurita* s.l., *Rhizostoma pulmo*, and *Pelagia noctiluca* was performed in autumn, winter and spring 2008/09. Jellyfish used in the experiment were collected by divers or from a boat using a plastic bag for each individual, which was put in a bucket with seawater and immediately transported to the laboratory. The wet weight was determined for each specimen before storing it at −30°C, without any additional washing. Before the experiment jellyfish biomass was homogenized with a sterilized blender, and one part was used in the enrichment experiment as an inoculum, while the rest was frozen and stored for elemental analysis.

The enrichment experiment was performed in spring 2009 during a jellyfish bloom in the Gulf of Trieste (northern Adriatic). Seawater for the experiment was collected with a Niskin sampler from 5 m depth at the offshore sampling station in the Gulf of Trieste (45° 32.804N; 13° 33.034E). Immediately after sampling, seawater was filtered through a 200 - µm mesh net and GF/F filters (Whatman Inc.) to remove particles and organisms larger than 0.8 µm. The filtrate was collected directly into four acid-washed, autoclaved polycarbonate Nalgen bottles. Equal amounts of jellyfish biomass was added (100 g w/w) into each of the enclosures, which were than marked as treatment A for *Aurelia*, P for *Pelagia* and R for *Rhizostoma*, to reach a final concentration of 12.5 g (w/w) per liter. Elemental analyses showed that the addition of 12.5 g (w/w) of jellyfish biomass per liter resulted in 162, 450 and 396 mg of carbon and 41, 123 and 106 mg of nitrogen in the A, P and R treatments, respectively. The C:N ratio was 4.6, 4.3 and 4.4 in the A, P and R treatments, respectively. An enclosure which contained only GF/F pre-filtered seawater served as a control (marked as treatment C). After the experimental set up, the enclosures were incubated *in situ* at 5 m depth in order to provide ambient temperature conditions, from 24th March until 2nd April 2009.

The first sampling was conducted immediately after inoculation (T0), then after 24 hours (T1), and afterwards on days 2, 3, 6 and 9 (T2, T3, T6, T9) of the experiment at approximately the same hour of the day. Samples were taken by pouring 500 ml of seawater from enclosures into acid-washed, autoclaved flasks. Samples for bacterial abundance, production and community structure together with samples for organic and inorganic matter analyses were taken each time.

### Bacterial Abundance and Production

Seawater samples for bacterial abundance were fixed with formaldehyde (<0.2 µm pre-filtered, 2% final concentration) and stored at 4°C. Samples were filtered onto 0.2 µm black polycarbonate filters (Millipore) and stained with DAPI (4’, 6-diamino-2-phenylindole, 1 µg mL^−1^, final, Sigma) [56]. DAPI-stained bacterial cells were counted using an epifluorescent microscope Olympus BX51. Randomly selected counting fields were photographed with a camera Image System DP70 and bacterial cells were counted manually. Bacterial carbon production (BCP) was measured using the ^3^H-leucine incorporation method (20 nM final concentration, PerkinElmer) employing the centrifugation protocol by Smith & Azam [57]. BCP was calculated as described by Simon & Azam [58].Bacterial growth rates (µ) were calculated from the slope in the exponential growth phase from the semilogarithmic graph (number of bacterial cells vs time).

### Chemical and Biochemical Analyses

The analyses of jellyfish elemental and biochemical compositions were performed on freeze-dried jellyfish homogenates. To avoid problems with high salt content, subsamples of homogenate were dialysed (MWCO 1000). Samples for particulate organic carbon (POC) and nitrogen (PN) analyses were collected by filtering seawater onto pre-combusted GF/F filters, which were stored at −20°C until analysis. Elemental composition (C-carbon and N-nitrogen) was determined from freeze-dried samples of jellyfish homogenate and filters using an Elemental Vario Micro Cube elemental analyzer (accuracy ± 0.01%). Total dissolved nitrogen (TDN), ammonium (NH_4_^+^), nitrite (NO_2_^−^), nitrate (NO_3_^−^), total dissolved phosphorus (TDP) and orthophosphate (PO_4_^3−^), were measured in GF/F pre-filtered subsamples using the standard protocols described in Parsons et al. [59]. Dissolved organic phosphorus (DOP) was calculated as the difference between TDP and PO_4_^3−^.

In order to estimate the total protein content of jellyfish, 10 mg of salt-free tissue sample was dissolved in 10 mL of lysis buffer (1X PBS, 0.5 mM EDTA, 0.1% Triton-X-100, 1% glycerol) and incubated at 95°C for 5 min. Afterward the sample was centrifuged at 10000×g for 15 min to remove debris. The total protein concentration of the supernatant was determined colorimetrically using a BCA kit according to the protocol supplied by the manufacturer (SIGMA) and quantified against a standard curve of BSA (bovine serum albumen) (concentration range 0.5–30 µg mL^−1^). The total protein concentration in tissue was expressed as % of salt - free dry weight. The dissolved protein concentrations were determined in seawater and in GF/F pre-filtered subsamples using the Bio-Rad Protein Assay Kit according to the standard Bradford method [60]. 2 mL triplicate aliquots of each subsample were centrifuged for 10 min at 20000×g at 4°C before analysis. OD_595_ was measured versus reagent blanks ( = 0.22 µm pre - filtered seawater) and protein concentrations were quantified against a standard curve of bovine serum albumen (concentration range 1–20 µg mL^−1^).

### DNA Extraction and PCR of 16S rRNA Genes from Bacterial Isolates

A defined volume of seawater sample was spread on ZoBell agar media and incubated in the dark at *in situ* temperature. Colonies with different morphologies were isolated throughout the experiment. After being clean streaked three times, a single colony of each isolate was inoculated into ZoBell liquid media and incubated in the dark at *in situ* temperature. Bacterial DNA was extracted using a Genomic DNA purification kit (Fermentas) or NucleoSpin Tissue kit (Macherey-Nagel) according to manufacturer protocol. Bacterial 16S rRNA genes were amplified using universal primers 27F and 1492R. The PCR reaction mix (50 µL) contained 1x reaction buffer (Tris KCl-MgCl_2_), 2 mM MgCl_2_, 0.2 mM dNTP, 1 µM of each primer, Taq polymerase (5 U µL^−1^, Fermentas) and 2 µL of DNA (50–100 ng). The PCR thermal cycler program was as follows: 94°C for 2 min; 30 cycles of 94°C for 1 min, 55°C for 1 min, 72°C for 2 min; and 72°C for 5 min. The size and quality of PCR products was confirmed by agarose gel electrophoresis. DNA concentration was measured fluorometrically using a Quant-It^TM^ dsDNA HS Assay kit (Invitrogen) and Qubit fluorometer (Invitrogen). The 16S rRNA genes were bidirectionally sequenced using 27F and 1492R primer with 23 ABI 3730XLs sequencer at Macrogen Inc. The quality of sequences was controlled by removing traces of sequencing primer using DNA baser (www.DNAbaser.com). Ambiguous base calls at the end of the sequences were also trimmed away. Database searches for sequence taxonomic identities were done using the genome Basic Local Alignment Search Tool (BLAST) at the National Centre for Biotechnology Information (NCBI) [61]. Sequences were deposited in GeneBank (NCBI) under the following accession numbers: JQ432567–JQ432570 from R treatment, JQ432571–JQ432575 from P treatment, JQ432576–JQ432580 from A treatment and JQ432581–JQ432587 from control.

### Extraction, PCR and DGGE Analysis of Total Bacterial Community DNA

At each sampling a defined volume of unfixed seawater from each enclosure was filtered onto 0.2 µm polyethersulfone membrane filters (47 mm diameter, PALL Inc.) for bacterial community DNA analysis. These filters were stored at −80°C until DNA extraction. DNA was extracted from the filters as described in Böstrom et al. [62], with slight modifications. DNA was precipitated at −20°C for 1 h, with 0.1 volume of sodium acetate (3 M NaAc, pH 5.2) and 0.6 volume of isopropanol. The pellet was washed with 70% ice-cold ethanol and dried in a speed- vac. Precipitated DNA was re-suspended in 0.02 µm pre-filtered, autoclaved TE buffer and kept at −20°C. Bacterial 16S rRNA were amplified using a universal primer 907R and 341F, with a 40 bp GC-clamp [63]. The PCR reaction mix (50 µL) contained 1x reaction buffer (Tris KCl-MgCl_2_), 2 mM MgCl_2_, 0.2 mM dNTP, 1 µM of each primer, Taq polymerase (5 U µL^−1^ Fermentas) and 2 µL of DNA (50–100 ng). The PCR touchdown protocol according to Don et al. [64] was used. The size, quality and concentration of PCR products was determined as described above. PCR products were analyzed by DGGE electrophoresis (C.B.S. Scientific Co.). 300 ng per lane of PCR product was loaded on 6% polyacrylamide gels (made from 40% acrylamide:N,N’-methylbisacrylamide 37.5:1, Sigma) containing a denaturant gradient top to bottom of 20 to 60% (100% denaturant is defined as 7 M urea (Sigma) and 40% (v/v) deionized formamide (Sigma)). Electrophoresis was run at 200V (35–40 mA) for 6 h using 1X TAE running buffer at 60°C. Gels were stained with 1x SYBR Gold nucleic acid stain (Invitrogen). Gels were visualized using a UV transilluminator. Using Image J software the presence, position and relative brightness of DGGE bands was visually detected and a similarity dendrogram was constructed from DGGE banding patterns using the Bray-Curtis coefficient and the group average was used as the linkage algorithm on square root-transformed data using Primer v5 software [65].

### Bacterial 16S rRNA Gene Clone Libraries and Phylogenetic Analyses

Bacterial 16S rRNA genes were amplified using the same DNA as for DGGE analysis and universal primers 27F and 1492R. The PCR reaction mixture (50 µL) was as described above. The PCR thermal cycler program was as follows: 95°C for 5 min; 30 cycles of: 95°C for 30 s, 50°C for 30 s, 72°C for 45 s, the last cycle was followed by a 7 min final incubation at an annealing temperature of 72°C to ensure that all the PCR products were 3’ adenylated. The size, quality and concentration of PCR products was determined as described above. Fresh PCR products were immediately ligated into a pCR® 2.1 commercially available vector (Invitrogen) and transformed using the heat shock principle into chemically competent *E.Coli* TOP 10 cells (Invitrogen) using a commercially available TA Cloning kit (Invitrogen) according to manufacturer protocol. The 16S rRNA gene inserts were partially sequenced using M13F primer with 23 ABI 3730XLs sequencer at Macrogen Inc. All sequences from 16S rDNA gene clone libraries were analyzed using the program Bellerophon (https://greengenes.lbl.gov/) to detect chimeric sequences. Detected chimeric sequences were removed. Database searches for sequence taxonomic identities were done using the genome Basic Local Alignment Search Tool (BLAST) at the National Center for Biotechnology Information (NCBI) [61]. Sequences were deposited in GeneBank (NCBI) under the following accession numbers: from JQ432588 to JQ432591 for Au9 clone library, from JQ432592 to JQ432608 for Pn9 clone library, from JQ432609 to JQ432644 for T0 clone library, from JQ432645 to JQ432651 for Rp6 clone library and from JQ432652 to JQ432710 for C9 clone library.

### Statistical Analyses

A two-way ANOVA with replication was performed on bacterial carbon production and a two-way ANOVA without replication was performed on bacterial abundance, protein and ammonium concentrations to assess differences between treatments and to detect temporal changes for each parameter. A Tukey HSD test of 95% confidence intervals was performed to compare individual treatments and to compare individual time points. All statistical analyses were performed using R statistical software (R Development Core Team, 2010).

## Supporting Information

Table S1 **A two - way ANOVA without replication was performed to assess difference in the bacterial abundance, protein and ammonium (NH_4_^+^) concentrations between jellyfish treatments (A, P, R) and the control (C) and to detect temporal changes for each of the parameters.**
(PDF)

Table S2 **A two - way ANOVA with replication was performed to assess difference in the bacterial carbon production among all treatments (A, P, R and C) and to detect temporal changes.**
(PDF)

Table S3 **16S rRNA gene clone libraries.** Bacterial clones from T0, C9, A9, P9 and R6 16S rRNA gene clone libraries from a jellyfish - enrichment experiment in 2009 in the Gulf of Trieste with their accession numbers. In the table there is also the name and an accession number of their closest relative in GeneBank (NCBI) with % of similarity, family, taxon and isolation source.
(PDF)
