# Supplementary material for: Jellyfish Modulate Bacterial Dynamic and Community Structure
Source: PLoS One. 2012 Jun 20;7(6):e39274. doi: 10.1371/journal.pone.0039274 (PMC3379990; doi:10.1371/journal.pone.0039274)
Supplement: Table S1 — A two - way ANOVA without replication was performed to assess difference in the bacterial abundance, protein and ammonium (NH4+) concentrations between jellyfish treatments (A, P, R) and the control (C) and to detect temporal changes for each of the parameters. (PDF) [file pone.0039274.s001.pdf]

Table S1. A two - way ANOVA without replication was performed to assess difference in the bacterial abundance, protein and ammonium (NH_4^+) concentrations between jellyfish treatments (A, P, R) and the control (**A**). A Tukey HSD test of 95% confidence intervals was performed to compare individual time points (**B**).

A) 2 - way ANOVA without replication

	**Df**	**F value**	**Pr(>F)**
**Abundance**			
Treatment	1	10.166	0.00657**
Day	5	7.489	0.00131**
Residuals	14		
**NH_4^+**			
Treatment	1	34.01	2.00E-05***
Day	5	3.811	0.0169*
Residuals	17		
**Proteins**			
Treatment	3	17.289	3.93E-05***
Day	5	1.411	0.276
Residuals	15		

B) Tukey HSD test - P value

**Day**	**Abundance**	**NH_4^+**	**Proteins**
1-0	0.999	0.999	0.947
2-0	0.993	0.999	0.999
3-0	0.249	0.996	0.992
6-0	0.010	0.107	0.809
9-0	0.010	0.217	0.879
2-1	0.999	0.999	0.813
3-1	0.376	0.981	0.709
6-1	0.017	0.072	0.323
9-1	0.017	0.152	0.401
3-2	0.527	0.961	0.999
6-2	0.029	0.056	0.945
9-2	0.029	0.121	0.975
6-3	0.401	0.238	0.980
9-3	0.400	0.430	0.993
9-6	1.000	0.998	0.999
