# Supplementary material for: Jellyfish Modulate Bacterial Dynamic and Community Structure
Source: PLoS One. 2012 Jun 20;7(6):e39274. doi: 10.1371/journal.pone.0039274 (PMC3379990; doi:10.1371/journal.pone.0039274)
Supplement: Table S2 — A two - way ANOVA with replication was performed to assess difference in the bacterial carbon production among all treatments (A, P, R and C) and to detect temporal changes. (PDF) [file pone.0039274.s002.pdf]

Table S2. A two - way ANOVA with replication was performed to assess difference in the bacterial carbon production among all treatments (A, P, R and C) (**A**). A Tukey HSD test of 95% confidence intervals was performed to compare individual treatments (**B**) and to compare individual time points (**C**).

A) 2 - way ANOVA with replication

	**Df**	**F value**	**Pr(>F)**
**Treatment**	3	46.59	7.52E-14***
**Day**	5	596.78	2.00E-16***
**Residuals**	45		

B) Tukey HSD test

**Treatment**	**P value**
A-C	0.000
P-C	0.000
R-C	0.000
P-A	0.075
R-A	0.897
R-P	0.299

C) Tukey HSD test

**Day**	**P value**
1-0	0.306
2-0	0.000
3-0	0.000
6-0	0.000
9-0	0.000
2-1	0.000
3-1	0.000
6-1	0.000
9-1	0.000
3-2	0.000
6-2	0.000
9-2	0.000
6-3	0.988
9-3	0.999
9-6	0.998
