# Supplementary material for: Jellyfish Modulate Bacterial Dynamic and Community Structure
Source: PLoS One. 2012 Jun 20;7(6):e39274. doi: 10.1371/journal.pone.0039274 (PMC3379990; doi:10.1371/journal.pone.0039274)
Supplement: Table S3 — 16S rRNA gene clone libraries. Bacterial clones from T0, C9, A9, P9 and R6 16S rRNA gene clone libraries from a jellyfish - enrichment experiment in 2009 in the Gulf of Trieste with their accession numbers. In the table there is also the name and an accession number of their closest relative in GeneBank (NCBI) with % of similarity, family, taxon and isolation source. (PDF) [file pone.0039274.s003.pdf]

Table S3: Bacterial clones from T0, C9, A9, P9 and R6 from 16S rRNA gene clone libraries from jellyfish-enrichment experiment in 2009 in the Gulf of Trieste with their accession numbers. In the table there is also a name and an accession number of the closest relative in GeneBank (NCBI) with % of similarity, family, taxon and isolation source.

Treatment	Clone	Acc. No.	Closest relative in GeneBank (NCBI), Acc. No.	Similarity	Family	Taxon	Source
T0	JELLYFISH_T0_A8	JQ432609	Uncultured bacterium clone 3C003027, EU801675	100%	SAR11	Alpha	Sea water
T0	JELLYFISH_T0_B3	JQ432610	Uncultured bacterium clone 6C232734, EU804774	99%	SAR11	Alpha	Sea water
T0	JELLYFISH_T0_B5	JQ432611	Uncultured alpha proteobacterium clone ARTE4_216, GU230208	100%	SAR11	Alpha	Sea water
T0	JELLYFISH_T0_B7	JQ432612	Uncultured Flavobacterium sp. TAI-2-81, AM259825	99%	Flavobacteriaceae	Flavo	Sea water
T0	JELLYFISH_T0_B8	JQ432613	Uncultured bacterium clone 3C003637, EU802209	100%	SAR11	Alpha	Sea water
T0	JELLYFISH_T0_B9	JQ432614	Uncultured alpha proteobacterium clone SHAG416, GQ348854	99%	SAR11	Alpha	Sea water
T0	JELLYFISH_T0_B11	JQ432615	Uncultured bacterium clone 3C003027, EU801675	100%	SAR11	Alpha	Sea water
T0	JELLYFISH_T0_C4	JQ432616	Uncultured bacterium clone 2C228151, EU800158	99%	SAR11	Alpha	Sea water
T0	JELLYFISH_T0_C5	JQ432617	Uncultured bacterium clone 1C226591, EU799038	99%	Rhodospirillaceae	Alpha	Sea water
T0	JELLYFISH_T0_C6	JQ432618	Uncultured alpha proteobacterium clone D13W_181, HM057775	99%	SAR11	Alpha	Sea water
T0	JELLYFISH_T0_C12	JQ432619	Uncultured bacterium clone 2C228148, EU800155	100%	SAR11	Alpha	Sea water
T0	JELLYFISH_T0_D1	JQ432620	Uncultured alpha proteobacterium clone SHAZ544, GQ349958	99%	SAR11	Alpha	Sea water
T0	JELLYFISH_T0_D2	JQ432621	Uncultured bacterium clone F9P1210_S_C08, HQ671782	99%		Cyano	Sea water
T0	JELLYFISH_T0_D4	JQ432622	Uncultured bacterium clone 1C226874, EU799293	99%		Gamma	Sea water
T0	JELLYFISH_T0_D8	JQ432623	Uncultured alpha proteobacterium clone A13W_1, HM057605	99%	SAR11	Alpha	Sea water
T0	JELLYFISH_T0_D10	JQ432624	Uncultured gamma proteobacterium HNA_I_100608-65, HM037612	99%		Gamma	Sea water
T0	JELLYFISH_T0_E1	JQ432625	Uncultured alpha proteobacterium clone ARTE1_97, GU230205	99%	SAR11	Alpha	Sea water
T0	JELLYFISH_T0_E4	JQ432626	Uncultured bacterium clone 6C232832, EU804865	99%	SAR11	Alpha	Sea water
T0	JELLYFISH_T0_E5	JQ432627	Uncultured alpha proteobacterium clone ARTE9_401, GU230237	99%	SAR11	Alpha	Sea water
T0	JELLYFISH_T0_E8	JQ432628	Uncultured bacterium clone 2C229509, EU801156	99%	Methylophilaceae	Beta	Sea water
T0	JELLYFISH_T0_E9	JQ432629	Uncultured bacterium clone 2C229516, EU801161	99%	SAR11	Alpha	Sea water
T0	JELLYFISH_T0_F5	JQ432630	Uncultured Flavobacterium sp. TAI-2-81, AM259825	99%	Flavobacteriaceae	Flavo	Sea water
T0	JELLYFISH_T0_F6	JQ432631	Uncultured alpha proteobacterium clone ARTE1_97, GU230205	99%	SAR11	Alpha	Spong
T0	JELLYFISH_T0_F7	JQ432632	Uncultured bacterium clone 3C003583, EU802165	99%	SAR11	Alpha	Sea water
T0	JELLYFISH_T0_F8	JQ432633	Uncultured alpha proteobacteria MB13F01, AY033325	99%		Alpha	Sea water
T0	JELLYFISH_T0_F9	JQ432634	Uncultured gamma proteobacterium clone SHAB429, GQ348549	99%		Gamma	Sea water
T0	JELLYFISH_T0_G3	JQ432635	Uncultured bacterium clone 2C228366, EU800317	98%	SAR11	Alpha	Sea water
T0	JELLYFISH_T0_G5	JQ432636	Uncultured alpha proteobacterium clone ARTE1_97, GU230205	99%	SAR11	Alpha	Sea water
T0	JELLYFISH_T0_G7	JQ432637	Uncultured bacterium clone 1C227739, EU800050	99%	SAR11	Alpha	Sea water
T0	JELLYFISH_T0_G10	JQ432638	Uncultured Flexibacter sp. clone TAU-7-69, AM259764	99%	Cytophagaceae	Flavo	Sea water
T0	JELLYFISH_T0_G11	JQ432639	Uncultured alpha proteobacterium clone ARTE1_97, GU230205	99%	SAR11	Alpha	Sea water
T0	JELLYFISH_T0_G12	JQ432640	Uncultured alpha proteobacterium clone D13W_181, HM057775	99%	SAR11	Alpha	Sea water
T0	JELLYFISH_T0_H2	JQ432641	Uncultured Flavobacteria bacterium Vis_St6_1272, FN433347	98%	Flavobacteriaceae	Flavo	Sea water
T0	JELLYFISH_T0_H5	JQ432642	Uncultured bacterium clone 3C003637, EU802209	100%	SAR11	Alpha	Sea water
T0	JELLYFISH_T0_H8	JQ432643	Uncultured bacterium clone 3C003583, EU802165	99%	SAR11	Alpha	Sea water
T0	JELLYFISH_T0_H11	JQ432644	Uncultured alpha proteobacterium clone ARTE1_210, GU230266	99%	SAR11	Alpha	Sea water
C9	JELLYFISH_C9_A1	JQ432652	Uncultured bacterium clone 1C227226, EU799607	99%	Flavobacteriaceae	Flavo	Sea water
C9	JELLYFISH_C9_A2	JQ432653	Uncultured Bacteroidetes bacterium clone D13W_13, HM057748	99%	Flavobacteriaceae	Flavo	Sea water

**C9**	JELLYFISH_C9_A3	JQ432654	Uncultured marine bacterium clone OS3BD103, JN233555	99%		Unclassifie	Sea water
**C9**	JELLYFISH_C9_A4	JQ432655	Uncultured bacterium clone W1-58, FJ545543	99%	Methylophilaceae	Beta	Sea water
**C9**	JELLYFISH_C9_A6	JQ432656	Uncultured gamma proteobacterium, FM958463	99%		Gamma	H. guttulatus
**C9**	JELLYFISH_C9_A7	JQ432657	Uncultured bacterium isolate lagoon october 2007, FN435246	99%	Alteromonadaceae	Gamma	Sea water
**C9**	JELLYFISH_C9_B1	JQ432658	Uncultured Flavobacteriaceae PEACE2006/69_P3, EU394565	98%	Flavobacteriaceae	Flavo	Sea water
**C9**	JELLYFISH_C9_B2	JQ432659	Uncultured Cryomorphaceae PEACE2006/31_P3, EU394563	99%	Flavobacteriaceae	Flavo	Sea water
**C9**	JELLYFISH_C9_B4	JQ432660	Uncultured Bacteroidetes bacterium clone D13W_13, HM057748	98%	Flavobacteriaceae	Flavo	Sea water
**C9**	JELLYFISH_C9_B5	JQ432661	Uncultured bacterium clone 1C227741, EU800052	99%	Rhodobacteraceae	Alpha	Sea water
**C9**	JELLYFISH_C9_B7	JQ432662	Uncultured bacterium isolate inlet june 2008, FN435240	99%		Gamma	Sea water
**C9**	JELLYFISH_C9_B9	JQ432663	Uncultured gamma proteobacterium clone 1, AM748176	99%		Gamma	Sea water
**C9**	JELLYFISH_C9_B10	JQ432664	Polaribacter dokdonensis strain MED152, DQ481463	99%	Flavobacteriaceae	Flavo	Sea water
**C9**	JELLYFISH_C9_B11	JQ432665	Marinomonas blandensis strain MED 121, DQ403809	98%	Oceanospirillaceae	Gamma	Sea water
**C9**	JELLYFISH_C9_B12	JQ432666	Uncultured bacterium clone 2C229430, EU801108	99%	SAR11	Alpha	Sea water
**C9**	JELLYFISH_C9_C2	JQ432667	Uncultured bacterium clone 1C227672, EU799992	99%	Rhodobacteraceae	Alpha	Sea water
**C9**	JELLYFISH_C9_C4	JQ432668	Polaribacter dokdonensis strain MED152, DQ481463	99%	Flavobacteriaceae	Flavo	Sea water
**C9**	JELLYFISH_C9_C5	JQ432669	Uncultured bacterium clone PH-C6, EF659435	99%	Rhodobacteraceae	Alpha	Sea water
**C9**	JELLYFISH_C9_C6	JQ432670	Uncultured bacterium clone W1-58, FJ545543	99%	Methylophilaceae	Beta	Sea water
**C9**	JELLYFISH_C9_C7	JQ432671	Uncultured bacterium clone 2C229430, EU801108	99%	SAR11	Alpha	Sea water
**C9**	JELLYFISH_C9_C8	JQ432672	Uncultured Rhodobacteraceae bacterium clone DS119, DQ234202	99%	Rhodobacteraceae	Alpha	Sea water
**C9**	JELLYFISH_C9_C10	JQ432673	Uncultured bacterium isolate inlet june 2008, FN435248	99%	Alteromonadaceae	Gamma	Sea water
**C9**	JELLYFISH_C9_C11	JQ432674	Uncultured bacterium clone 1C227214, EU799596	99%	Rhodobacteraceae	Alpha	Sea water
**C9**	JELLYFISH_C9_C12	JQ432675	Uncultured bacterium isolate inlet june 2008, FN435248	99%	Alteromonadaceae	Gamma	Sea water
**C9**	JELLYFISH_C9_D1	JQ432676	Marinomonas blandensis strain MED 121, DQ403809	98%	Oceanospirillaceae	Gamma	Sea water
**C9**	JELLYFISH_C9_D2	JQ432677	Uncultured alpha proteobacterium clone M0-Ar2-P4C08, EF016462	99%	Rhodobacteraceae	Alpha	Sea water
**C9**	JELLYFISH_C9_D4	JQ432678	Uncultured bacterium clone 2C229430, EU801108	99%	SAR11	Alpha	Sea water
**C9**	JELLYFISH_C9_D5	JQ432679	Vibrio splendidus isolate PB1-10rrnA, EU091325	99%	Vibrionaceae	Gamma	Atlantic halibut fry
**C9**	JELLYFISH_C9_D7	JQ432680	Uncultured Bacteroidetes bacterium clone M0-Ar2-P4F11, EF016482	100%	Flavobacteriaceae	Flavo	Sea water
**C9**	JELLYFISH_C9_D10	JQ432681	Uncultured bacterium clone 1C226862, EU799281	98%	SAR11	Alpha	Sea water
**C9**	JELLYFISH_C9_D12	JQ432682	Uncultured marine microorganism clone 4035AA_68, EU187996	99%	SAR11	Alpha	Sea water
**C9**	JELLYFISH_C9_E2	JQ432683	Uncultured Bacteroidetes bacterium clone C8W_85, HM057654	99%	Flavobacteriaceae	Flavo	Sea water
**C9**	JELLYFISH_C9_E6	JQ432684	Uncultured Bacteroidetes bacterium clone D13W_13, HM057748	99%	Flavobacteriaceae	Flavo	Sea water
**C9**	JELLYFISH_C9_E7	JQ432685	Uncultured Alteromonas sp. clone F3C21, AY936195	99%	Alteromonadaceae	Gamma	Sea water
**C9**	JELLYFISH_C9_E8	JQ432686	Uncultured bacterium isolate inlet june 2008, FN435248	99%	Alteromonadaceae	Gamma	Sea water
**C9**	JELLYFISH_C9_E9	JQ432687	Uncultured bacterium clone 1C227710, EU800023	99%	Rhodobacteraceae	Alpha	Sea water
**C9**	JELLYFISH_C9_E10	JQ432688	Uncultured Bacteroidetes bacterium clone F3C31, AY794159	99%	Flavobacteriaceae	Flavo	Sea water
**C9**	JELLYFISH_C9_E12	JQ432689	Uncultured bacterium clone 1C227018, EU799422	99%		Gamma	Sea water
**C9**	JELLYFISH_C9_F3	JQ432690	Uncultured Comamonadaceae bacterium clone DS173, DQ234255	99%	Comamonadaceae	Beta	Sea water
**C9**	JELLYFISH_C9_F4	JQ432691	Uncultured bacterium clone 3C003501, EU802098	99%	SAR11	Alpha	Sea water
**C9**	JELLYFISH_C9_F5	JQ432692	Uncultured alpha proteobacterium clone C8W_100, HM057657	99%	Rhodobacteraceae	Alpha	Sea water
**C9**	JELLYFISH_C9_F6	JQ432693	Uncultured bacterium clone 1C226600, EU799046	99%	Methylophilaceae	Beta	Sea water
**C9**	JELLYFISH_C9_F8	JQ432694	Uncultured bacterium clone 1C226805, EU799228	99%		Gamma	Sea water
**C9**	JELLYFISH_C9_F10	JQ432695	Uncultured bacterium clone 6C232873, EU804900	99%	SAR11	Alpha	Sea water
**C9**	JELLYFISH_C9_G1	JQ432696	Uncultured bacterium isolate inlet june 2008, FN435241	99%	Alteromonadaceae	Gamma	Sea water
**C9**	JELLYFISH_C9_G2	JQ432697	Pseudoalteromonas sp. 'A1 isolate-2', EF474129	100%	Pseudoalteromonada	Gamma	Sea water
**C9**	JELLYFISH_C9_G3	JQ432698	Uncultured bacterium clone ZL1708, EU268118	98%		Cyano	Sea water
**C9**	JELLYFISH_C9_G6	JQ432699	Uncultured bacterium isolate inlet june 2008, FN435248	99%	Alteromonadaceae	Gamma	Sea water
**C9**	JELLYFISH_C9_G7	JQ432700	Uncultured bacterium clone 1C226833, EU799255	99%	Methylophilaceae	Beta	Sea water

**C9**	JELLYFISH_C9_G8	JQ432701	Rhodobacteraceae bacterium IMCC1933, GQ468664	99%	Rhodobacteraceae	Alpha	Sea water
**C9**	JELLYFISH_C9_G10	JQ432702	Uncultured bacterium clone W2-57, FJ545632	99%	SAR11	Alpha	Sea water
**C9**	JELLYFISH_C9_G12	JQ432703	Uncultured bacterium isolate inlet june 2008, FN435240	99%		Gamma	Sea water
**C9**	JELLYFISH_C9_H1	JQ432704	Uncultured prasinophyte clone OM5, U70715	99%		Cyano	Sea water
**C9**	JELLYFISH_C9_H5	JQ432705	Uncultured SAR86 cluster gamma proteobacterium clone 45, AM748220	99%	SAR86	Gamma	Sea water
**C9**	JELLYFISH_C9_H6	JQ432706	Uncultured bacterium clone 3C003594, EU802174	96%		Gamma	Sea water
**C9**	JELLYFISH_C9_H7	JQ432707	Uncultured Bacteroidetes bacterium clone D13W_13, HM057748	98%	Flavobacteriaceae	Flavo	Sea water
**C9**	JELLYFISH_C9_H8	JQ432708	Uncultured phototrophic eukaryote clone HE17, AY702176	98%		Cyano	Sea water
**C9**	JELLYFISH_C9_H9	JQ432709	Uncultured bacterium clone ZL1708, EU268118	99%		Cyano	Sea water
**C9**	JELLYFISH_C9_H10	JQ432710	Rhodobacteraceae bacterium IMCC1933, GQ468664	99%	Rhodobacteraceae	Alpha	Sea water
**A9**	JELLYFISH_Au9_A8	JQ432588	Vibrio sp. Da4, AF242272	99%	Vibrionaceae	Gamma	Sea urchin
**A9**	JELLYFISH_Au9_B6	JQ432589	Vibrio tasmaniensis strain Mj28, GQ455006	99%	Vibrionaceae	Gamma	Spider crab
**A9**	JELLYFISH_Au9_C1	JQ432590	Flavobacteriaceae bacterium G121102s2_3, AY353820	98%	Flavobacteriaceae	Flavo	Diatom detritus
**A9**	JELLYFISH_Au9_D1	JQ432591	Vibrio splendidus isolate PB1-10rrnJ, EU091334	99%	Vibrionaceae	Gamma	Atlantic halibut fry
**P9**	JELLYFISH_Pn9_A1	JQ432592	Shewanella kaireitica c931, AB094598	98%	Shewanellaceae	Gamma	Sea sediment
**P9**	JELLYFISH_Pn9_A5	JQ432593	Pseudoalteromonas sp. 520P1, AB196167	99%	Pseudoalteromonada	Gamma	Sea water
**P9**	JELLYFISH_Pn9_A7	JQ432594	Lacinutrix sp. MEBiC01653, EU581705	98%	Flavobacteriaceae	Flavo	Sea sediment/ water
**P9**	JELLYFISH_Pn9_A8	JQ432595	Shewanella fidelia strain KMM3589, AF420313	99%	Shewanellaceae	Gamma	Sea sediment
**P9**	JELLYFISH_Pn9_A10	JQ432596	Vibrio splendidus isolate PB1-10rrnE, EU091329	99%	Vibrionaceae	Gamma	Atlantic halibut fry
**P9**	JELLYFISH_Pn9_B1	JQ432597	Pseudoalteromonas sp. 520P1, AB196167	100%	Pseudoalteromonada	Gamma	Sea water
**P9**	JELLYFISH_Pn9_B3	JQ432598	Bizionia sp. NF1-21, FJ889663	98%	Flavobacteriaceae	Flavo	Sea sediment
**P9**	JELLYFISH_Pn9_B10	JQ432599	Pseudoalteromonas sp. BSs20130, EU365507	98%	Pseudoalteromonada	Gamma	Sea sediment
**P9**	JELLYFISH_Pn9_B11	JQ432600	Pseudoalteromonas sp. 520P1, AB196167	97%	Pseudoalteromonada	Gamma	Sea sediment
**P9**	JELLYFISH_Pn9_C1	JQ432601	Bizionia sp. NF1-21, FJ889663	98%	Flavobacteriaceae	Flavo	Sea sediment
**P9**	JELLYFISH_Pn9_C3	JQ432602	Shewanella sp. B246, FN295778	99%	Shewanellaceae	Gamma	Bryozoa
**P9**	JELLYFISH_Pn9_C5	JQ432603	Bizionia sp. NF1-21, FJ889663	98%	Flavobacteriaceae	Flavo	Sea sediment
**P9**	JELLYFISH_Pn9_C8	JQ432604	Pseudoalteromonas sp. BSw20106, EU365604	98%	Pseudoalteromonada	Gamma	Sea water
**P9**	JELLYFISH_Pn9_D2	JQ432605	Pseudoalteromonas denitrificans strain MAR_121_B08_2010-01-20, [illegible]	97%	Pseudoalteromonada	Gamma	Sea water
**P9**	JELLYFISH_Pn9_D10	JQ432606	Shewanella fidelia strain KMM3589, AF420313	99%	Shewanellaceae	Gamma	Sea sediment
**P9**	JELLYFISH_Pn9_D11	JQ432607	Uncultured gamma proteobacterium clone PM1-19, EF215793	99%	Oceanospirillaceae	Gamma	Artifical surface in sea [illegible]
**P9**	JELLYFISH_Pn9_D12	JQ432608	Bizionia sp. NF1-21, FJ889663	98%	Flavobacteriaceae	Flavo	Sea sediment
**R6**	JELLYFISH_Rp6_E1	JQ432645	Pseudoalteromonas sp. B199b, FN295769	99%	Pseudoalteromonada	Gamma	Bryozoa
**R6**	JELLYFISH_Rp6_E7	JQ432646	Pseudoalteromonas sp. 12, DQ642815	99%	Pseudoalteromonada	Gamma	Laminaria japonica
**R6**	JELLYFISH_Rp6_F5	JQ432647	Uncultured Vibrionaceae bacterium clone MS-C128, FJ949311	99%	Vibrionaceae	Gamma	Calcareous sandy [illegible]
**R6**	JELLYFISH_Rp6_F10	JQ432648	Pseudoalteromonas sp. SM0524, EU548075	99%	Pseudoalteromonada	Gamma	Decaying brown alge
**R6**	JELLYFISH_Rp6_G1	JQ432649	Pseudoalteromonas sp. BSw20040, EU365563	100%	Pseudoalteromonada	Gamma	Sea water
**R6**	JELLYFISH_Rp6_G3	JQ432650	Pseudoalteromonas sp. 520P1, AB196167	100%	Pseudoalteromonada	Gamma	Unknown
**R6**	JELLYFISH_Rp6_H10	JQ432651	Pseudoalteromonas sp. BCw097, FJ889597	100%	Pseudoalteromonada	Gamma	Sea water
